# AN UNUSUAL CAUSE OF ACUTE ABDOMEN: SPLENIC INFARCTION

**DOI:** 10.1590/0102-6720201700040015

**Published:** 2017

**Authors:** Itai GHERSIN, Adi LEIBA

**Affiliations:** 1 Medical Corps, Israeli Defense Force, Tel-Hashomer, Ramat-Gan, Israel

**Keywords:** Abdomen, acute, Lymphoma, Spleen, Abdome agudo, Linfoma, Baço.

## INTRODUCTION

Splenic infarction is an uncommon cause of acute abdomen^4^. Causes include hematologic disease (such as leukemia, lymphoma, myelofibrosis, polycythemia vera), thromboembolic disorders, splenic vascular disease, pancreatic disorders, vasculitis, portal hypertension, bacterial endocarditis, sickle cell disease and infiltrative disorders^1^ ^,^ ^3^. It is a well-documented complication of lymphoma. However, there are only a few reports of splenic infarction as the initial lymphomas manifestation^2^ ^,^ ^5^, with none describing an acute abdomen due to splenic infarct as the initial presentation of diffuse large B-cell lymphoma (DLBCL) to our knowledge. 

Herein, is reported a case of a patient who presented with acute abdomen, and was found to have splenic infarction on imaging. Subsequent investigation revealed DLBCL as the etiology of splenic infarction.

## CASE REPORT

A 36 year old previously healthy male military officer presented to his primary care clinic with a three day history of epigastric pain. The pain was accompanied by nausea, without vomiting, and by anorexia. His vital signs were normal. He was ill-appearing and distressed, with abdominal examination showing marked tenderness in the epigastrium with focal peritoneal signs, making clinical assessment for organomegaly impossible.

Suspecting a surgical abdominal emergency, the primary care physician referred the patient to the emergency room. There, after evaluation by internist and surgeon, urgent laboratory tests and a chest and abdomen CT were performed. Complete blood count showed anemia (hemoglobin-10 g/dl), leukocytosis (12.3*10^9^/l) with a normal differential and normal platelets. Blood chemistry revealed normal electrolytes and renal function, elevated liver enzymes (AST-210 U/l, ALP-551 U/l), LDH-13,000 U/l and uric acid 10mg/dl. Screening tests of coagulation failed to demonstrate any abnormalities.

**FIGURE 1:**
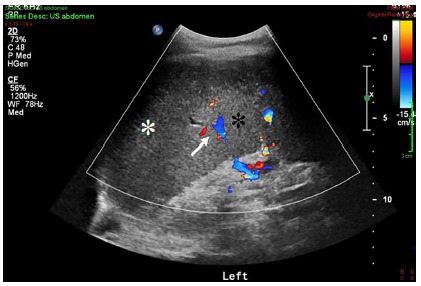
Longitudinal color Doppler scan of the spleen demonstrating an upper pole peripheral hypoechoic region, which lacks blood flow on color Doppler (white asterix). It is sharply demarcated from the normal, more echogenic splenic parenchyma (black asterix), which demonstrates normal color Doppler flow in the intraparenchymal splenic vessels (white arrow)

Abdominal sonography revealed splenomegaly (splenic diameter of 22 cm) with a large hypoechoic and heterogenic area at the spleen’s periphery, without blood flow within this area (Figure 1), a finding consistent with a large splenic infarct.

**FIGURE 2:**
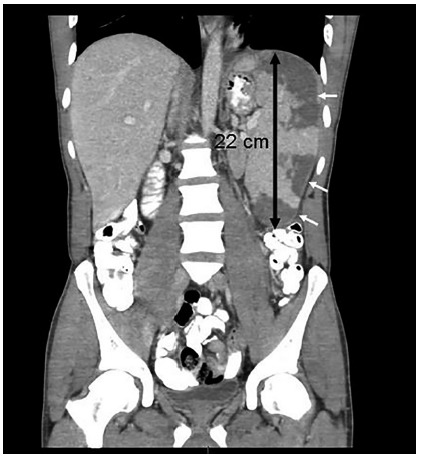
Coronal multiplanar reformat from a contrast enhanced abdominal CT, demonstrates a markedly enlarged spleen with a span of 22 cm (black arrow with measurement), with multiple peripheral well-defined wedge-shaped hypodensities, highly suggestive of multiple splenic infarcts (white arrows).

A chest+abdominal CT was performed, revealing marked hepatomegaly and splenomegaly (22 cm) with multiple peripheral well-defined wedge-shaped hypodense splenic lesions, highly suggestive of multiple splenic infarcts (Figure 2). Significant lymph node enlargement both above and below the diaphragm was demonstrated as well. Left sided pleural effusion was also noted.

Based on these findings, a diagnosis of lymphoma, complicated by splenic infarcts, was suggested. Further analysis, including lymph node biopsy, bone marrow biopsy with immunophenotyping (CD20 positive, CD5 weakly positive, KI67=95%), and peripheral blood lymphocyte immunophenotyping (CD5, CD19 positive, CD23 negative), revealed the patient was suffering from a stage IV diffuse large B-cell lymphoma. Treatment with hydration, allopurinol and analgesics was immediately initiated, with relatively rapid improvement of abdominal symptoms, and was followed by chemotherapy with Hyper-CVAD regimen. 

The patient initially underwent autologous peripheral stem cell transplant, but has suffered from disease recurrence following the treatment. Later he achieved remission following allogeneic bone marrow transplantation, and has now returned to serve in the military in an administrative role. 

## DISCUSSION

This case serves as a reminder that lymphomas can initially manifest as an acute abdomen due to splenic infarction. We believe it is the first case describing an acute abdomen due to splenic infarct as the initial presentation of diffuse large B-cell lymphoma (DLBCL) in English medical literature.

Although it is a rather rare form of presentation, physicians from all specialties need to be aware of it, as prompt evaluation, diagnosis and initiation of treatment are of utmost importance in such cases. Surgeons should be particularly alert to it, as most cases of acute abdomen undergo a surgical evaluation in the emergency department. 
